# Hypertension prevalence, awareness, treatment, and control and predicted 10-year CVD risk: a cross-sectional study of seven communities in East and West Africa (SevenCEWA)

**DOI:** 10.1186/s12889-020-09829-5

**Published:** 2020-11-13

**Authors:** Samson Okello, Alfa Muhihi, Shukri F. Mohamed, Soter Ameh, Caleb Ochimana, Abayomi Olabayo Oluwasanu, Oladimeji Akeem Bolarinwa, Nelson Sewankambo, Goodarz Danaei

**Affiliations:** 1 Department of Internal Medicine, Mbarara University of Science and Technology, P. O Box 1410, Mbarara, Uganda; 2 Lown Scholars Program, Department of Global Health and Population, Harvard T.H. Chan School of Public Health, Boston, MA USA; 3 Division of Infectious Diseases and International Health, Department of Medicine, University of Virginia Health Systems, Charlottesville, VA USA; 4 Africa Academy for Public Health, Dar es Salaam, Tanzania; 5 Department of Community Health, Muhimbili University of Health and Allied Sciences, Dar es Salaam, Tanzania; 6 Health and Systems for Health Unit, African Population and Health Research Center (APHRC), Nairobi, Kenya; 7 Department of Community Medicine, Faculty of Medicine, College of Medical Sciences, University of Calabar, Calabar, Cross River State Nigeria; 8 Ochimana Caleb Foundation, Federal Capital Territory, Abuja, Nigeria; 9 University Health Services, University of Ibadan, Ibadan, Nigeria; 10 Department of Epidemiology and Community Health, University of Ilorin, Ilorin, Nigeria; 11 College of Health Sciences, Makerere University College of Health Sciences, Kampala, Uganda; 12 Department of Global Health and Population, Department of Epidemiology, Harvard T.H. Chan School of Public Health, Boston, MA USA

**Keywords:** Hypertension epidemiology and management, Predicted 10-year CVD risk, East and West Africa

## Abstract

**Background:**

Few studies have characterized the epidemiology and management of hypertension across several communities with comparable methodologies in sub-Saharan Africa. We assessed prevalence, awareness, treatment, and control of hypertension and predicted 10-year cardiovascular disease risk across seven sites in East and West Africa.

**Methods:**

Between June and August 2018, we conducted household surveys among adults aged 18 years and above in 7 communities in Kenya, Nigeria, Tanzania, and Uganda. Following a standardized protocol, we collected data on socio-demographics, health insurance, and healthcare utilization; and measured blood pressure using digital blood pressure monitors. We estimated the 10-year cardiovascular disease (CVD) risk using a country-specific risk score and fitted hierarchical models to identify determinants of hypertension prevalence, awareness, and treatment.

**Results:**

We analyzed data of 3549 participants. The mean age was 39·7 years (SD 15·4), 60·5% of whom were women, 9·6% had ever smoked cigarettes, and 32·7% were overweight/obese. A quarter of the participants (25·4%) had hypertension, more than a half of whom (57·2%) were aware that they had diagnosed hypertension. Among those diagnosed, 50·5% were taking medication, and among those taking medication 47·3% had controlled blood pressure. After adjusting for other determinants, older age was associated with increased hypertension prevalence, awareness, and treatment whereas primary education was associated with lower hypertension prevalence. Health insurance was associated with lower hypertension prevalence and higher chances of treatment. Median predicted 10-yr CVD risk across sites was 4·9% (Interquartile range (IQR), 2·4%, 10·3%) and 13·2% had predicted 10-year CVD risk of 20% or greater while 7·1% had predicted 10-year CVD risk of > 30%.

**Conclusion:**

In seven communities in east and west Africa, a quarter of participants had hypertension, about 40% were unaware, half of those aware were treated, and half of those treated had controlled blood pressure. The 10-year predicted CVD risk was low across sites. Access to health insurance is needed to improve awareness, treatment, and control of hypertension in sub-Saharan Africa.

## Background

For the past few decades, the burden of hypertension has shifted from high-income countries to low- and middle-income countries including sub-Saharan Africa (SSA) [1]. In Africa, the estimated number of people with hypertension has increased steadily from 54·6 million in 1990 to 92·3 million in 2000 (70% rise) and 130·2 million in 2010 (41% increase from the year 2000). It is projected to rise to 216·8 million by the year 2030 (66% rise from the year 2010) [2]. .Hypertension is widespread in SSA, with Tanzania and Kenya experiencing the highest prevalence [3, 4]. The high burden of hypertension in SSA has severe consequences including increased risk for morbidity and mortality from cardiovascular disease (stroke, myocardial infarction, and hypertensive heart diseases) [5].

Results from the Prospective Urban Rural Epidemiology (PURE) study indicate that low-income countries have the lowest rates for awareness, treatment, and control of hypertension globally [6]. In Africa, sub-Saharan Africa compared to North African countries have low levels of awareness, treatment, and control of hypertension especially in rural areas [7]. Low awareness and poor control of hypertension in SSA have been attributed to poor health infrastructure and compliance to treatment, with poverty partly the underlying cause [8].

A recent analysis of data from the WHO Stepwise Approach to Surveillance (STEPS) has shown poor hypertension care in sub-Saharan Africa. Countries in this region have the worst hypertension care cascade performance relative to their predicted performance based on Gross Domestic Product (GDP) per capita [9]. However, the surveys were conducted more than 5 years ago, for example, 2014 in Uganda and 2012 in Tanzania. In addition, several other countries, including Nigeria and Kenya, were not included as they don’t have a recent STEPS survey. Moreover, this study did not include a previous diagnosis of hypertension in their definition of hypertension and no estimates of hypertension control [9].

With the above limitations, the studies characterizing the epidemiology of hypertension across several communities with comparable methodologies in sub-Saharan Africa are scarce. Yet understanding the magnitude, awareness, treatment, and control of hypertension in SSA is key to inform appropriate and cost-effective preventive and control strategies. Therefore, we assessed prevalence, awareness, treatment, and control of hypertension in Kenya, Tanzania, Uganda, and Nigeria.

## Methods

### Study design

This descriptive multi-site cross-sectional study was conducted among 3675 adults aged 18 years and above from seven communities in four countries in Tanzania, Uganda, Kenya, and Nigeria. The study settings comprised rural areas in Nigeria (Olorunda Abaa in Oyo state, Ogane-Uge in Kogi state, and Okpok Ikpa in Cross River State); semi-urban (Ikire town in Osun state Nigeria and Ukonga ward in Dar es Salam in Tanzania); and urban communities (Soroti municipality in Uganda and Viwandani slum of Nairobi in Kenya).

### Study populations and sampling procedures

Participants were recruited using a representative sample from each community. In Kenya, participants were randomly selected from the Nairobi Urban Health and Demographic Surveillance System (NUHDSS) registry. A list of potential participants was collected from the NUHDSS and the inclusion and exclusion criteria were applied. Finally, we randomly selected 300 participants from the list of potential participants.

Similarly, participants from Nigerian sites were selected using random sampling techniques. In Okpok Ikpa site, a house-to-house survey of adults was performed in the rural areas of Okpok Ikpa, Odukpani LGA, Cross River State, south-south region of Nigeria. In Ogane Uge site in Nigeria, we selected a random sample of households from rural areas of Ogane-Uge, Oganenigwu, Dekina L.G.A, all in Kogi State. In Olorunda Abaa of Oyo State, participants were selected from a random sample of households. In Ikire site, we conducted a household survey among adults in Ikire, Irewole LGA, Osun state, a semi-urban community in South West Nigeria.

In Uganda, participants were sampled from all divisions of Soroti municipality. Starting at a landmark such as church/mosque or school, and selected every third household to the right of the main entrance to the landmark. The first sampled household was the initiator of the sample in that area and sequentially sampled every third household on the right of the main entrance of the previous household until the sample was achieved.

In Tanzania, participants were selected by simple random sampling from a list of households of Ukonga ward, Ilala municipal area, Dar es Salaam region in the Dar es Salaam Health and Demographic Surveillance System (HDSS).

Participants were adults aged 18 years or greater residing in the area of study. Pregnant women and individuals with physical impairments preventing measurement of blood pressure or body weight and height were excluded. A resident was defined as someone who has stayed within the area for at least 3 months and is expecting to stay for another 3 months. If there were more than one eligible participant in a household, we used the Kish method [10] to select one of them. In the event that a selected individual was not home at the time of the visit, 3 attempts on separate days, including evenings on week days and weekends were made before sampling another eligible household member. If a selected household had no eligible individual, we visited the immediate neighboring household until an eligible participant was found.

### Data collection procedures

Trained research assistants conducted data collection using a structured standardized questionnaire to collect information on socio-demographic and economic (asset ownership) characteristics of the participants [11]. We also collected information on common risk factors for non-communicable diseases (NCDs) including tobacco and alcohol use, history of diagnosis and/or management of cardiovascular disease and its risk factors (hypertension, diabetes mellitus, dyslipidemia), and a list of current medications.

### Measurements

#### Blood pressure

Blood pressure was measured on the left upper arm using a digital blood pressure machine, with patient in a seated position after 3–5 min of rest. Three systolic blood pressure (SBP) and diastolic blood pressure (DBP) measurements were taken at least 5 min apart using portable sphygmomanometers (OMRON-Healthcare-Co HEM-7211-E-Model-M6; Kyoto, Japan). The mean of the second and third readings was used in this analysis. Hypertension was defined as average SBP ≥140 mmHg and/or DBP ≥90 mmHg and/or self-report of previous diagnosis with or without current treatment with antihypertensive medications in accordance with the Seventh Report of the Joint National Committee on Prevention, Detection, Evaluation and Treatment of High Blood Pressure [12]. Treatment of hypertension was defined as current or prior (those whose medication ran-out) use of antihypertensive medication. Among those treated, control was defined as having systolic blood pressure below 140 mmHg and diastolic blood pressure below 90 mmHg. We intentionally avoided using the 2017 American Heart Association (AHA) and American College of Cardiology (ACC) definition of hypertension as it would significantly increase the number considered hypertensive and the current national guidelines in these countries have not yet incorporated these new lower thresholds. We defined hypertension awareness as a self-report of ever diagnosis of hypertension by a healthcare provider.

#### Anthropometric measurements

Weight and height were taken with the participant wearing light clothing and with no shoes using the standardized scales (seca 762, Hanover, USA) and height using a roll-up measuring stadiometers (seca 206, Hanover, USA). Body weight was measured and recorded to the nearest 0·1 kg and height was measured and recorded to the nearest 0·1 cm. Body mass index (BMI) was then calculated as body weight per height squared (kg/m^2^). Overweight was defined as BMI ≥25 kg/m^2^ but < 30 kg/m^2^ and obesity as BMI ≥30 kg/m^2^ [13]. Waist and hip circumferences were measured to the nearest 0.1 cm (using seca tape measure) using the standard methods [14].

#### Socioeconomic characteristics

Data on ownership of household items such as radio, television, telephone, sofa, refrigerator, bicycle, car, and having working electricity; house ownership, construction materials (floor, walls and roofing materials); source of fuel for cooking and lighting; source of water supply for home use and drinking; and sanitation facility were also collected.

#### Other covariates

Sociodemographic information including age, gender, marital status, education level, and occupation were collected. Marital status was grouped into never married, married or living together, divorced or separated, and widowed. Educational level attainment was categorized according to the highest level reached in primary school, secondary school, or tertiary education (including vocational training). We collected occupation data in pre-coded categories: self-employed, government employee, private employer, and unemployed.

### Statistical analyses

We estimated the prevalence of hypertension for all participants and by site, and hypertension awareness, treatment, and control of hypertension among those with a prior diagnosis of hypertension. We used principle component analysis to generate an assets ownership index score based on household utilities and assets to derive composite measures with highest discriminatory capabilities [15]. Participants were divided into quintiles of these scores (poorest, poor, fair, rich, and richest) [11].

We examined association between prevalence, awareness, treatment, and control of hypertension with a-prior set of covariates: age (continuous), gender (men and women), employment (unemployed, government, and private), health insurance (yes or no), education (primary school and below, secondary school, and tertiary education), alcohol use (yes or no), current smoker (yes or no), and diabetes (yes or no).

We used hierarchical models with a logit link function and communities (sites) as random intercepts, to identify both individual and community characteristics independently associated with mean systolic blood pressure after adjusting for age, marital status, highest level of education attained, smoking, alcohol use (Model 1); employment status, body mass index (Model 2), and additionally adjusted for health insurance (Model 3). The models with prevalence as outcome are for all participants; those of awareness are among those with hypertension; those for treatment are among those who were aware; and those for control are for those on treatment.

We computed standardized rates by employing direct standardization to the World Health Organization Standard Population age-structure for the period 2000–2025 [16] using 10-year age bands. These allows for the calculation of standardized rates that are comparable across regions and time [16]. The overall rates by site indicate the rate that would result if all populations had the same age distribution [17].

We used the Globorisk score [18] to predict the 10-year risk of a first fatal and non-fatal cardiovascular disease (CVD) (stroke and coronary heart disease) for adults aged 40 or greater for each site. The office-based Globorisk score is a country-specific CVD risk prediction model that estimates the 10-year risk of a first fatal and non-fatal stroke and ischemic heart disease, based on age (years), gender, systolic blood pressure (mm Hg), body mass index (BMI), and smoking status (yes/no) [18]. We considered two different thresholds to define high risk for future cardiovascular disease: > 20% risk scores on the basis of the WHO guidelines [19] and 30% as the threshold on the basis of the global NCD target [20]. Participants with a score < 7·5% were considered low-risk. We used boxplots to compare predicted CVD risks for each site for men and women who were categorized as low-risk or high-risk. All analyses were complete case analyses performed using Stata version 15·1 (Stata Corp., TX, USA).

## Results

A total 3675 participants were enrolled at seven study sites over the period June to August 2018 with an overall response rate of 91% (79% in Viwandani an urban slum in Nairobi, Kenya and 100% in Ikire and Ogane-Uge both rural areas in Nigeria). Of these, we excluded 109 participants who had no or implausible blood pressure measurements and 17 who were missing weight and height measurements. The sample analyzed constituted 3549 participants with a mean age of 39·7 years (SD 15·4), out of which 60·5% were women. Participants in Nigerian sites on average were older than those from East African sites (*p* < 0·0001 for difference in mean age) (Table 1).

**Table 1:** Baseline characteristics, SevenCEWA study 2018

Characteristic	All (*n* = 3549)	Ikire, Nigeria (*n* = 489)	Ogane-Uge, Nigeria (*n* = 403)	Okpok Ikpa, Nigeria (*n* = 465)	Olorunda Abaa, Nigeria (*n* = 708)	Soroti, Uganda (*n* = 760)	Ukonga, Tanzania (*n* = 424)	Viwandani, Kenya (*n* = 300)
*Setting*		Semi-urban	Rural	Rural	Rural	Urban	Semi-urban	Urban
*Demographics*								
Age (years), mean (SD)	39·7 (15·4)	48·1 (18·1)	39·2 (19·5)	38·5 (14·0)	41·2 (12·9)	33·7 (12·4)	43·9 (14·1)	34·7 (10·9)
Women, n (%)	2147 (60·5)	269 (55·0)	211 (52·4)	225 (48·4)	458 (64·7)	534 (70·3)	305 (71·9)	145 (48·3)
Asset index								
Poorest	646 (18·2)	100 (20·5)	88 (21·8)	99 (21·3)	135 (19·1)	154 (20·3)	89 (20·9)	63 (21·0)
Poor	614 (17·3)	98 (20·0)	81 (20·2)	97 (20·9)	130 (18·4)	153 (20·1)	80 (18·8)	57 (19·0)
Fair	742 (20·9)	98 (20·0)	166 (41·2)	131 (28·1)	135 (19·1)	152 (20·0)	82 (19·3)	60 (20·0)
Rich	507 (14·3)	95 (19·5)	–	58 (12·5)	135 (19·1)	154 (20·3)	87 (20·5)	60 (20·0)
Richest	575 (16·2)	98 (20·0)	53 (13·1)	80 (17·2)	133 (18·7)	147 (19·3)	86 (20·28)	60 (20·0)
Highest level of Education attained								
None	456 (12·8)	102 (20·9)	105 (26·1)	105 (22·6)	46 (6·5)	72 (9·5)	22 (5·2)	4 (1·3)
Primary	1217 (34·3)	115 (23·5)	153 (38·0)	207 (44·5)	154 (21·8)	198 (26·1)	270 (63·7)	120 (40·0)
Secondary	1279 (36·0)	193 (39·5)	123 (30·5)	117 (25·2)	301 (42·5)	284 (37·4)	105 (24·8)	156 (52·0)
Tertiary	580 (16·3)	68 (13·9)	22 (5·5)	32 (6·9)	205 (29·0)	206 (27·1)	27 (6·4)	20 (6·7)
Employment status								
Self-employed	2121 (59·8)	395 (80·8)	307 (76·2)	309 (66·5)	560 (79·1)	222 (29·2)	255 (60·1)	73 (24·3)
Government employee	302 (8·5)	21 (4·3)	8 (2·0)	26 (5·6)	63 (8·9)	91 (12·0)	28 (6·6)	65 (21·7)
Private employer	333 (9·4)	31 (6·3)	23 (5·7)	38 (8·2)	47 (6·6)	56 (7·4)	13 (3·1)	125 (41·7)
Unemployed	785 (22·1)	42 (8·6)	65 (16·1)	84 (18·1)	38 (5·4)	391 (51·4)	128 (30·2)	37 (12·3)
Refused to answer	8 (0·2)	–	–	8 (1·7)	–	–	–	–
Smoking, n (%)								
Never	3036 (85·5)	439 (89·8)	221 (54·8)	389 (83·7)	672 (94·9)	718 (94·5)	375 (88·4)	222 (74·0)
Ever	409 (11·5)	47 (9·6)	114 (28·3)	71 (15·3)	31 (4·4)	42 (5·5)	26 (6·1)	78 (26·0)
Declined to answer	104 (2·9)	3 (0·6)	68 (16·9)	5 (1·1)	5 (0·7)	–	23 (5·4)	–
Al1cohol use								
Never	2690 (75·8)	427 (87·3)	226 (56·1)	256 (55·1)	672 (94·9)	633 (83·3)	349 (82·3)	127 (42·3)
Ever	727 (20·5)	44 (9·0)	90 (22·3)	187 (40·2)	31 (4·4)	127 (16·7)	75 (17·7)	173 (57·7)
Declined to answer	132 (3·7)	18 (3·7)	87 (21·6)	22 (4·7)	5 (0·7)	–	–	–
Self-reported Diabetes mellitus, n (%)	86 (2·4)	25 (5·1)	10 (2·5)	1 (0·2)	20 (2·8)	8 (1·1)	19 (4·5)	3 (1·0)
*Measurements*								
Body mass index category, n (%)								
Underweight (< 18·5 kg/m^2^)	349 (9·8)	54 (11·0)	91 (22·6)	2 (0·4)	56 (7·9)	106 (13·9)	23 (5·4)	17 (5·7)
Normal (18·5 to < 25 kg/m^2^)	1918 (54·0)	264 (54·0)	194 (48·1)	347 (74·6)	362 (51·1)	449 (59·1)	133 (31·4)	169 (56·3)
Overweight/Obese (> 25 kg/m^2^)	1159 (32·7)	139 (28·4)	110 (27·3)	110 (23·7)	250 (35·3)	181 (23·8)	256 (60·4)	113 (37·7)
Missing	123 (3·5)	32 (6·5)	8 (2·0)	6 (1·3)	40 (5·6)	24 (3·2)	12 (2·8)	1 (0·3)
Waist circumference (both genders)								
Men (≥ 102 cm)^a^	101 (2·8)	16 (3·3)	8 (2·0)	5 (1·1)	16 (2·3)	14 (1·8)	41 (9·7)	1 (0·3)
Women (≥ 88 cm)^a^	865 (24·4)	104 (21·3)	48 (11·9)	127 (27·3)	211 (29·8)	156 (20·5)	180 (42·5)	39 (13·0)
Blood pressure								
Mean SBP (mmHg), mean (SD)^^^	122·9 (20·8)	128·5 (23·9)	126·2 (20·5)	126·4 (19·6)	119·1 (21·6)	122·8 (17·2)	125·3 (21·3)	110·0 (16·3)
Mean DBP (mmHg), mean (SD)^^^	77·9 (12·8)	82·0 (14·0)	77·3 (12·9)	80·3 (12·4)	73·8 (12·6)	80·0 (11·2)	77·2 (12·7)	73·4 (11·4)

Out of the total sample, the sites contributed as follows: 13·8% from Ikire, 11·4% from Ogane-Uge, 13·1% from Okpok Ikpa, 19·9% from Olorunda Abaa, 21·4% from Soroti, 11·9% from Ukonga, and 8·5% from Viwandani. A total 55 participants declined to respond to asset ownership questions 15 (3·7%) in Ogane-Uge and 40 (5·6%) in Olorunda Abaa, Nigeria

Across sites, 44% of participants lived in rural areas of Ogane-Uge (11·4%), Okpok Ikpa (13·1%), and Olorunda Abaa (19·9%) all in Nigeria, a quarter lived in semi-urban areas [Ikire, Nigeria (13·8%) and Ukonga, Tanzania (11·9%)], and 29·9% lived in urban communities in Soroti, Uganda (21·4%) and Viwandani, Kenya (8·5%). Participants in Ukonga, Tanzania had the highest prevalence of obesity (60%) whereas those in Ogane-Uge, Nigeria had the highest prevalence of underweight (22%) (Table 1).

Overall, 25·4, 95% Confidence Interval (CI) (23·7%, 26·6%) of participants had hypertension. Nigerian communities had the highest crude prevalence of hypertension i.e., 38·6, 95%CI (34·2%, 43·0%) in Ikire, 33·0, 95%CI (28·4%, 37·7%) in Ogane-Uge, 23·3, 95%CI (20·3%, 26·6%) in Olorunda Abaa, and 20·4, 95%CI (17·9%, 25·6%) in Okpok Ikpa. Among the three East African sites, Ukonga in Tanzania had the highest crude prevalence at 28·5, 95%CI (24·3%, 33·1%) followed by Soroti in Uganda with 20·4, 95%CI (17·6%, 23·4%) and the lowest crude prevalence was recorded in Viwandani in Kenya with a 9·7, 95%CI (6·6%, 13·6%) (Table 2).

**Table 2:** Crude and age-standardized group-specific prevalence rates of hypertension, SevenCEWA study 2018

Characteristic	Crude prevalence rate	Standardized prevalence rate (95% CI)
Gender		
Male	24.8	25.2 (23.0, 27.3)
Female	25.2	24.8 (23.1, 26.5)
Age (in years)		
15–20	12.5	12.3 (5.8, 18.8)
20–29	10.4	10.4 (8.4, 12.4)
30–39	17.5	17.5 (15.1, 19.9)
40–49	28.4	28.4 (25.0, 31.7)
50–59	35.9	36.0 (31.2, 40.8)
> 60	58.5	58.7 (54.2, 63.2)
Site		
Ikire, Nigeria	38.6 (34.2, 43.0)	27.5 (24.6, 30.4)
Ogane-Uge, Nigeria	33.0 (28.4, 37.7)	27.7 (24.4, 30.9)
Okpok, Nigeria	20.4 (17.9, 25.6)	12.6 (11.2, 14.1)
Olorunda Abaa, Nigeria	23.3 (20.3, 26.6)	20.8 (18.4, 23.2)
Soroti, Uganda	20.4 (17.6, 23.4)	26.9 (23.9, 29.8)
Ukonga, Tanzania	28.5 (24.3, 33.1)	23.0 (19.7, 26.3)
Viwandani, Kenya	9.7 (6.6, 13.6)	6.6 (4.9, 8.3)

The age-standardized prevalence of hypertension was 16·3, 95%CI (14·5, 18·1) for women and 15·6, 95%CI (13·5, 17·6) for men. When stratified by site, the age-standardized prevalence was highest in Ogane-Uge, Nigeria at 22·1, 95%CI (18·0, 26·1) and lowest in Viwandani, Kenya at 11·3, 95%CI (7·4, 15·1) (Table 2).

Among the 901 participants with hypertension, 43·1, 95%CI (39·8%, 46·4%) were not aware that they had hypertension. Of those who were knew that they had hypertension, 49·5, 95%CI (42·9%, 51·7%) were not taking medications, and of those taking medication 52·7, 95%CI (44·4%, 56·7%) did not have their blood pressure controlled.

**Fig. 1:**
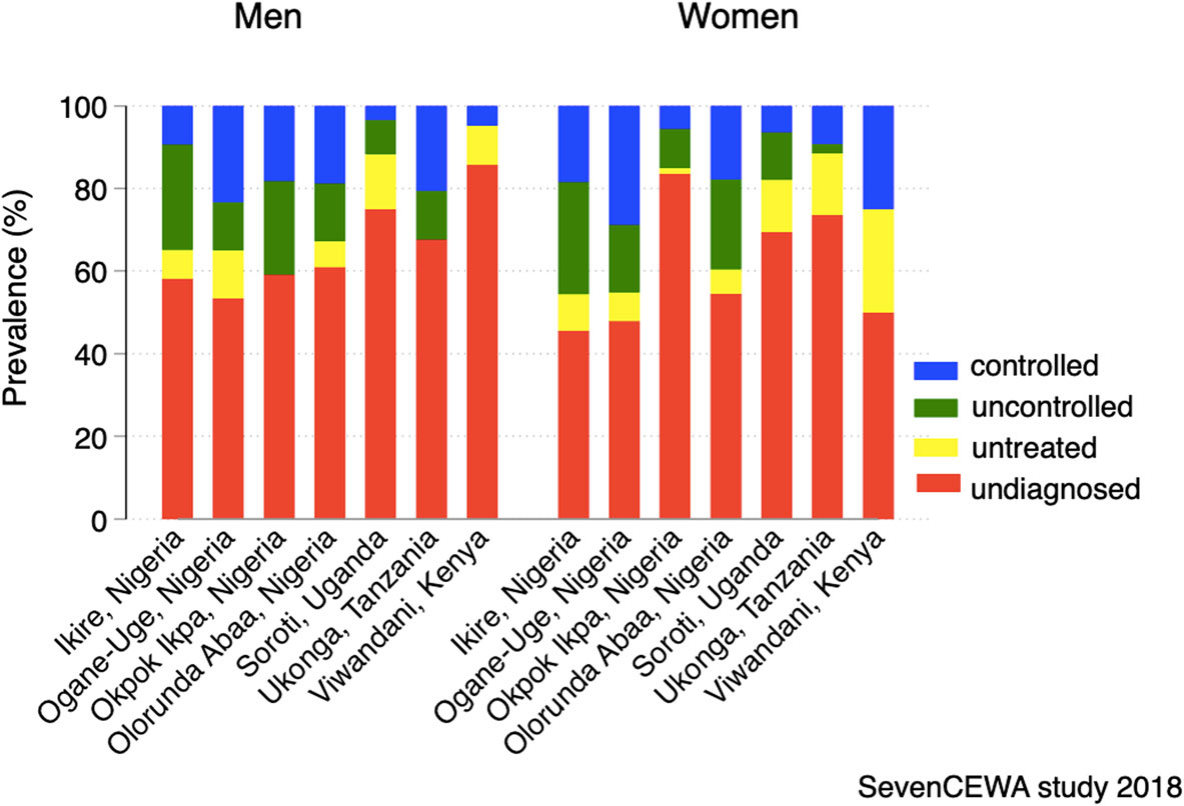
Proportions of awareness, treatment, and control of hypertension by gender and site, SevenCEWA study 2018

Despite the low prevalence of hypertension in Viwandani (Kenya), about three-quarters [75·9, 95%CI 56·5%, 89·7%] of those with elevated blood pressures were not aware that they had hypertension. On the contrary, Nigerian study sites with higher prevalence of hypertension had comparatively higher proportions of awareness of hypertension compared with sites in Tanzania and Kenya (Fig. 1).

Compared with participants of other sites, participants from Soroti, Uganda and Okpok Ikpa, Nigeria had higher rates of diagnosed but untreated hypertension; 78·9, 95%CI (70·3%, 86·0%) and 70·5, 95%CI (60·3%, 79·4%) of those diagnosed, respectively. Overall study sites in Nigeria had higher blood pressure control rates compared to those in east Africa (Fig. 1).

In models adjusted by site, for both gender, the factors associated with higher mean systolic blood pressures were older age and being overweight/obese. In contrast, being privately employed (compared with unemployed) among both genders, and among women having attained any education (compared with no education) were associated with lower mean systolic blood pressure (Table 3) Paradoxically, among men current smoking compared to never smoking was associated with a lower mean systolic blood pressure (− 4·2 mmHg, 95% CI -7·5, − 0·9).

**Table 3:** Association between systolic blood pressures (mmHg followed by 95% confidence interval) and sociodemographic, lifestyle and healthcare variables, adjusted by site, SevenCEWA study 2018

Characteristic	Women (*n* = 2147)			Men (*n* = 1402)		
	Model 1	Model 2	Model 3	Model 1	Model 2	Model 3
Age (per 10 years)	4·8 (4·0, 5·6)	5·1 (4·3, 5·8)	4·6 (3·7, 5·4)	3·5 (2·6, 4·5)	3·3 (2·3, 4·2)	3·3 (2·4, 4·2)
Marital Status						
Single	3·7 (0·7, 6·7)	5·4 (2·5, 8·3)	3·3 (0·2, 6·4)	1·8 (−1·7, 5·3)	1·1 (−2·4, 4·7)	0·9 (− 2·7, 4·5)
Married	Ref	Ref	Ref	Ref	Ref	Ref
Separated	−3·7 (−7·6, 0·1)	3·0 (0·1, 6·0)	− 3·6 (−7·4, 0·2)	−5·5 (− 10·5, −0·5)	−4·5 (−9·8, 0·9)	−4·6 (−9·9, 0·7)
Widowed	0·5 (− 2·9, 3·9)	4·0 (1·1, 6·9)	0·8 (− 2·7, 4·4)	4·8 (− 3·4, 12·9)	5·1 (− 3·1, 13·3)	4·0 (− 4·3, 12·4)
Highest level of Education attained						
None	Ref	Ref	Ref	Ref	Ref	Ref
Primary	−3·6 (−6·8, −0·4)	−1·5 (−4·8, 1·7)	−3·3 (−6·7, − 0·1)	1·2 (− 3·6, 6·1)	−1·0 (− 6·2, 4·3)	−0·8 (− 6·1, 4·5)
Secondary	− 6·5 (− 9·9, − 3·1)	−3·6 (− 6·9, − 0·3)	−6·0 (− 9·6, − 2·5)	−1·6 (− 6·7, 3·5)	−3·9 (− 9·4, 1·5)	−3·9 (− 9·4, 1·6)
Tertiary	−6·7 (− 10·4, − 3·0)	−3·0 (− 6·8, 0·7)	−5·0 (− 9·0, − 1·1)	−1·2 (− 6·6, 4·2)	− 2·8 (−8·5, 3·0)	−2·5 (− 8·3, 3·3)
Smoking, n (%)						
Never	Ref	Ref	Ref	Ref	Ref	Ref
Ever	2·4 (− 3·5, 8·3)	2·7 (− 3·3, 8·7)	3·8 (−2·2, 9·7)	− 5·1 (−8·1, − 2·1)	− 4·2 (− 7·5, − 0·9)	− 4·2 (− 7·5, − 0·9)
Alcohol use						
Never	Ref	Ref	Ref	Ref	Ref	Ref
Ever	0·9 (− 2·3, 4·1)	− 0·4 (− 3·6, 2·8)	1·1 (− 2·1, 4·3)	− 1·6 (− 4·5, 1·4)	− 0·3 (− 3·3, 2·7)	−0·2 (− 3·2, 2·9)
Employment status						
Self-employed		Ref	Ref		Ref	Ref
Government		−1·3 (−4·8, 2·1)	−0·8 (− 4·7, 3·1)		− 1·1 (− 4·9, 2·6)	−0·7 (− 4·7, 3·4)
Private employer		−4·4 (−7·8, − 1·0)	−4·8 (− 8·7, − 0·9)		− 3·6 (− 7·2, − 0·1)	−3·5 (− 7·1, 0·1)
Unemployed		2·6 (0·6, 4·7)	2·5 (0·4, 4·6)		1·5 (− 1·8, 4·8)	1·9 (−1·5, 5·3)
Body mass index category, n (%)						
Underweight (< 18·5 kg/m^2^)		Ref	Ref		Ref	Ref
Normal (18·5 to < 25 kg/m^2^)		1·5 (−1·7, 4·6)	0·3 (− 2·9, 3·5)		5·7 (2·2, 9·2)	6·0 (2·5, 9·6)
Overweight/Obese (> 25 kg/m^2^)		6·6 (3·4, 9·8)	4·4 (1·1, 7·7)		8·5 (4·4, 12·6)	9·0 (4·9, 13·1)
Health insurance						
Uninsured			Ref			Ref
Insured			−2·9 (−5·8, 0·1)			−1·0 (−4·8, 2·7)

In multivariable analyses, each 10-year increase in age for both sexes was associated with higher odds of prevalent hypertension (adjusted Odds Ratio 1·4, 95%CI 1·4, 1·5), whereas attainment of any education (versus no education) and having health insurance (aOR 0·6, 95%CI 0·5, 0·8) were associated with lower prevalence of hypertension particularly among women (Table 4) Older age was also associated with a higher odds of hypertension awareness for both sexes (aOR 1·2, 95%CI 1·1, 1·3) and primary education was associated with lower odds of awareness among women (aOR 0·5, 95%CI 0·3, 0·7) (Table 4) Finally, older age was also associated with higher odds of treatment for both sexes (aOR 1·2, 95%CI 1·1, 1·3). Having health insurance was also associated with a higher chance of being treated among women (aOR 1·5, 95%CI 1·2, 1·9) (Table 4).

**Table 4:** Associations of hypertension prevalence, awareness, and treatment for all participants and by gender (adjusted odds ratios and 95% confidence interval), SevenCEWA study 2018

	Both genders				Women				Men			
	Prevalence	Awareness	Treatment	Control	Prevalence	Awareness	Treatment	Control	Prevalence	Awareness	Treatment	Control
Number of participants (number with outcome) (%)	901 (25·4)	515 (57·2)	260 (50·5)	123 (47·3)	551 (25·7)	343 (62·3)	160 (46·6)	76 (47·5)	350 (25·0)	172 (49·1)	100 (58·1)	47 (47·0)
Age (each 10 yrs)	1·4 (1·4, 1·5)	1·2 (1·1, 1·3)	1·2 (1·1, 1·3)	0·7 (0·6, 0·8)	1·4 (1·3, 1·5)	1·2 (1·1, 1·3)	1·2 (1·0, 1·4)	0·7 (0·6, 0·9)	1·5 (1·3, 1·6)	1·2 (1·1, 1·4)	1·1 (0·9, 1·3)	0·6 (0·5, 0·8)
Level of Education												
None	Ref	Ref	Ref	Ref	Ref	Ref	Ref	Ref	Ref	Ref	Ref	Ref
Primary	0·6 (0·5, 0·8)	0·5 (0·3, 0·7)	0·6 (0·4, 0·9)	0·4 (0·2, 1·0)	0·6 (0·4, 0·7)	0·4 (0·3, 0·6)	0·5 (0·3, 0·8)	0·5 (0·2, 1·1)	0·7 (0·4, 1·2)	0·5 (0·3, 1·1)	0·9 (0·4, 2·3)	0·3 (0·1, 1·1)
Secondary	0·6 (0·4, 0·8)	0·7 (0·5, 1·0)	1·0 (0·6, 1·6)	0·4 (0·2, 0·9)	0·6 (0·4, 0·9)	0·7 (0·4, 1·1)	0·9 (0·5, 1·7)	0·4 (0·2, 0·9)	0·6 (0·4, 1·1)	0·6 (0·3, 1·3)	1·2 (0·5, 3·0)	0·5 (0·1, 2·1)
Tertiary	0·7 (0·5, 0·9)	0·8 (0·5, 1·2)	1·0 (0·6, 1·8)	0·3 (0·1, 0·6)	0·6 (0·4, 0·8)	0·8 (0·5, 1·4)	1·5 (0·7, 3·1)	0·3 (0·1, 0·9)	0·9 (0·5, 1·6)	0·7 (0·3, 1·6)	0·8 (0·3, 2·2)	0·2 (0·1, 1·3)
Wealth index												
Poorest	0·8 (0·7, 1·1)	0·7 (0·5, 1·0)	1·1 (0·7, 1·7)	1·4 (1·7, 2·7)	0·9 (0·7, 1·3)	0·8 (0·5, 1·2)	1·1 (0·6, 1·9)	0·9 (0·4, 2·1)	0·7 (0·5, 1·1)	0·6 (0·3, 1·1)	1·0 (0·5, 2·2)	3·5 (1·0, 12·9)
Poor	0·9 (0·7, 1·1)	0·9 (0·6, 1·2)	0·8 (0·5, 1·4)	1·1 (0·5, 2·5)	1·0 (0·7, 1·3)	0·9 (0·6, 1·4)	0·6 (0·3, 1·1)	1·2 (0·5, 3·0)	0·7 (0·5, 1·0)	0·8 (0·5, 1·5)	1·7 (0·8, 3·8)	1·1 (0·2, 4·7)
Fair	Ref	Ref	Ref	Ref	Ref	Ref	Ref	Ref	Ref	Ref	Ref	Ref
Rich	0·8 (0·7, 1·1)	0·8 (0·6, 1·2)	0·9 (0·6, 1·5)	1·2 (0·5, 2·5)	0·7 (0·5, 1·0)	0·8 (0·5, 1·3)	0·7 (0·3, 1·3)	0·7 (0·3, 2·0)	1·0 (0·7, 1·4)	0·8 (0·4, 1·4)	1·5 (0·7, 3·1)	1·7 (0·4, 6·9)
Richest	0·9 (0·7, 1·2)	0·9 (0·6 1·3)	1·0 (0·6, 1·6)	1·4 (0·6, 3·0)	0·8 (0·5, 1·1)	0·8 (0·5, 1·4)	0·8 (0·4, 1·6)	1·2 (0·5, 3·2)	1·1 (0·8, 1·6)	0·9 (0·5, 1·5)	1·3 (0·6, 2·8)	1·6 (0·4, 6·6)
Health Insurance												
Uninsured	Ref	Ref	Ref	Ref	Ref	Ref	Ref	Ref	Ref	Ref	Ref	Ref
Insured	0·6 (0·5, 0·8)	0·8 (0·6, 1·1)	1·6 (1·4, 1·9)	0·7 (0·4, 1·5)	0·7 (0·5, 0·9)	0·9 (0·5, 1·4)	1·5 (1·2, 1·9)	0·8 (0·3, 2·1)	0·6 (0·4, 0·8)	0·7 (0·4, 1·2)	0·8 (0·4, 1·6)	0·6 (0·2, 1·7)

^a^Adjustment was done for study site and gender

Although we had smaller numbers and larger uncertainties for analysis of controlled blood pressure as outcome, each decade increase in age was associated with lower odds of control (aOR 0·7, 95%CI 0·6 to 0·8) (Table 4).

**Fig. 2:**
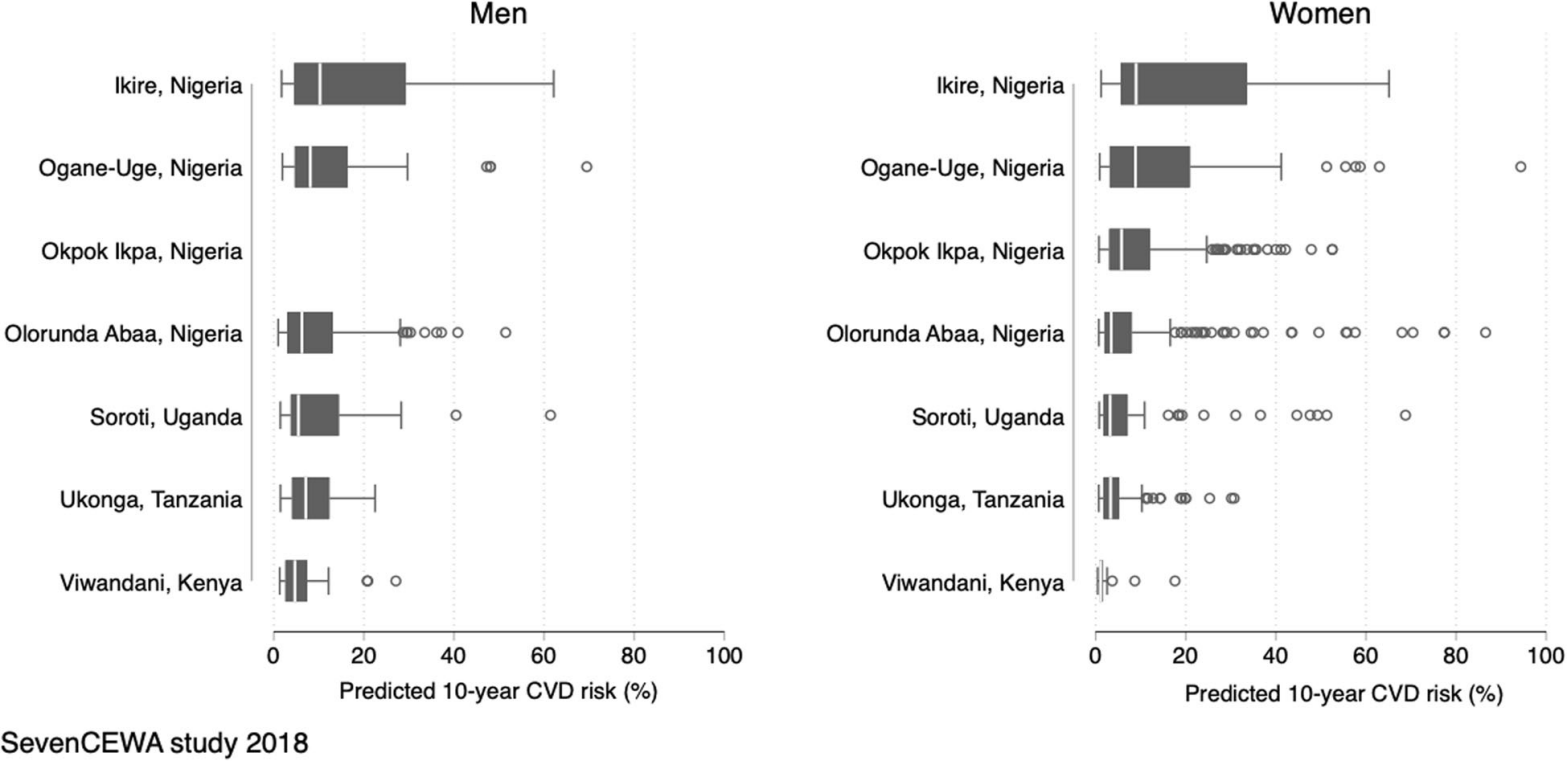
Predicted 10-year risk of a first fatal and non-fatal CVD for adults aged 40 or greater by gender and site using the Globorisk equations for each country [16], SevenCEWA study 2018. Footnote: Box plots represent 25th, median, and 75th percentiles of predicted 10-year CVD risk by gender and site. We excluded Okpok Ikpa site in this analysis of men because it had only 7 men aged > 40 yrs. thus the unstable were estimates

For participants aged 40 years and older, the overall median predicted 10-yr CVD risk of a first fatal and non-fatal CVD (stroke and ischemic heart disease) across all sites was fairly low at 4·9% IQR (2·4%, 10·3%) i.e., for men median 6·5% (IQR 3·7%, 13·1%) and women 3·9% (IQR 1·9%, 8·9%). We excluded men in Okpok Ikpa site because only 7 men aged > 40 yrs. were enrolled which would give unstable estimates. Noteworthy, the 10-year risk of CVD varied substantially across sites with highest risks estimated in Ikire, Nigeria for both men (median 10·3%, IQR 4·5%, 29·3%) and women (median 9·0%, IQR 5·6%, 33·6%). The lowest predicted 10-yr CVD risk for both gender were in Viwandani, Kenya for men (median 4·7%, IQR 2·6%, 7·4%) and women (median 1·2%, IQR 0·8%, 1·6%) (Fig. 2).

**Fig. 3:**
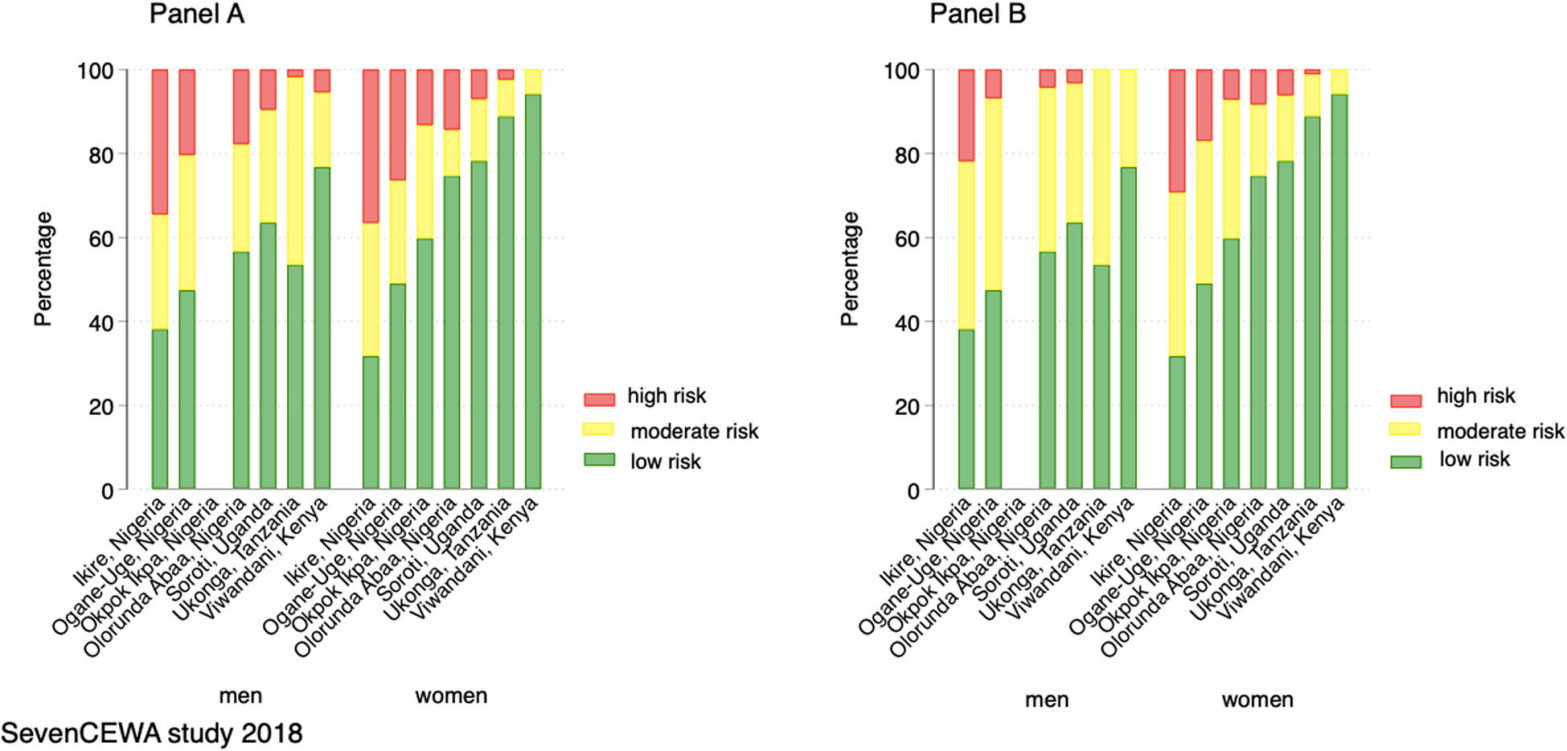
Predicted 10-year risk of first fatal and non-fatal CVD for adults aged 40 or greater by gender and site using the Globorisk equations for each country [1] by WHO threshold of > 30% high risk (Panel **a**) and Global NCD target of 30% as high risk (Panel **b**). SevenCEWA study 2018

Overall 13 % (13·2%) had predicted 10-yr CVD risk of 20% or greater as per the WHO guidelines [19] and 7·1% had predicted 10 year CVD risk using the 30% as the threshold of the global NCD target [20] (Fig. 3**).**

## Discussion

Prevalence, awareness, and blood pressure control of hypertension at the seven study sites in East and West Africa varied substantially. Other than documentation of these differences, our results may help to fully understand how hypertension affects sub-Saharan African countries as well as highlight the need to customize awareness, treatment, and prevention approaches according to the needs of each community and country. Such information is essential to the design of effective interventions aimed at minimizing rising rates of hypertension and it’s complications [21]. Overall, 43% of participants with hypertension were not aware. Similar to other studies of hypertension in sub-Saharan Africa, we found hypertension unawareness was more common among men [3, 22]. These data are consistent with a review on hypertension in SSA, which found levels of awareness under 40% for both sexes [5, 9].

Among participants with prevalent hypertension, 13% had their blood pressure controlled. This proportion is quite low, in part due to low levels of awareness. Also, 3 of 10 participants who were aware of their status received treatment, which could indicate a low level of engagement with primary health care providers and the cost of treatment poses a challenge in accessing treatment.

Although several prior studies have found health insurance associated with treatment for hypertension and blood pressure control [23–25], they largely been conducted in resource rich countries with established health insurance coverage unlike the current study settings were a dismal number were on health insurance.

Taken together, the poor control of hypertension is representative of the systemic issues facing the delivery of essential chronic care such as the socioeconomic determinants of hypertension, barriers to treatment, the inadequacy of healthcare infrastructure, the low levels of trained health care personnel, and adherence [26]. To achieve higher coverage of hypertension awareness and blood pressure control requires strengthening of the primary care system in particular provision of universal health insurance as well as outreach and community based approaches, to ensure effective screening, adherence and follow up, development and implementation of guidelines for use by primary care personnel, and enhance access to essential medicines [27–29]. Use of mobile health approaches are low-hanging fruits in sub-Saharan Africa that could increase health care delivery given the penetration of mobile phones in the region [30].

Of note, the finding of lower systolic blood pressure among current smokers compared to never smokers in men is in keeping with findings by others [31–33]. Some biologically plausible explanations for the lower blood pressure in smokers have been proposed including tolerance to continuous nicotine exposure and adaptations to the pressor effect of nicotine after chronic exposure [34]. In addition, the lower blood pressure could be an effect of the vasodilatory effects of cotinine (a major metabolite of nicotine with a half-life of about 12 h) [35]. However, conflicting evidence exist on the long term effects of smoking on blood pressure with some cohort studies reporting lower blood pressures [32, 34] and others reporting higher blood pressure among smokers compared to non-smokers [36]. There is a potential effect modification by age and gender and duration of smoking. It is beyond the scope of this study ascertain whether the prospective relationship between smoking and blood pressure.

The predicted 10-year risk of fatal and non-fatal CVD disease (stroke and ischemic heart disease) was low. Overall, the population under study was of a relatively younger age (mean age 40 years), had low rates of self-reported diabetes and smoking—key factors in the Globorisk prediction model [18]. There is no evidence on the comparability of existing risk algorithms in identifying high-risk individuals among sub-Saharan African populations, as such we could not compare our results with any. Prior studies used CVD risk prediction equations that were not country-specific thus did not captured the national differences in CVD rates [37].

Our study has several strengths. We studied diverse African communities using similar standard and validated questionnaires and measurement protocols to collect information on many lifestyle and socioeconomic factors which greatly reduces the potential for misclassification bias. However, our results should be interpreted with some limitations in mind. First, there is a possibility of unmeasured confounding as in any other observational study. Second, all participants were of African ancestry and there were no rural communities sampled in the East Africa. Therefore, our findings should not be extrapolated to other ethnicities and rural communities in East Africa. Third, the predicted 10-year CVD risk might be underestimated due to under-reporting of smoking because of social desirability bias. Finally, population-based surveys are subject to the healthy volunteer bias [38], thus leading to underestimation of the hypertension proportions.

## Conclusions

In conclusion, we observed high prevalence, low awareness, treatment and control of hypertension in seven communities in East and West Africa. Overall, the predicted 10-year CVD risk was low despite sex-specific and region-specific differences. Our data show stark sex-specific and region-specific differences that will require further detailed understanding to inform effective intervention strategies. Moreover, given the low levels of awareness of hypertension, and the related consequences of hypertension control, universal health insurance coupled with improvements in health promotion and system strengthening could help improve awareness, treatment, and control of hypertension in sub-Saharan Africa.

## Data Availability

The dataset used and/or analysed during the current study is available from the corresponding author on reasonable request.

## Abbreviations

BMI: Body mass index
CVD: Cardiovascular Disease
DBP: Diastolic Blood Pressure
GDP: Gross Domestic Product
NCD: Non-communicable diseases
SBP: Systolic Blood Pressure
SevenCEWA: Seven Communities in East and West Africa
SSA: Sub-Saharan Africa
WHO STEPS: World health Organization Stepwise Approach to Surveillance
